# Supplementary material for: Cold-Pressed Oilseed Cakes as Alternative and Sustainable Feed Ingredients: A Review
Source: Foods. 2023 Jan 17;12(3):432. doi: 10.3390/foods12030432 (PMC9914438; doi:10.3390/foods12030432)
Supplement: Supplementary file 1 [file foods-12-00432-s001.zip › Supplementary table S1.pdf]

Supplementary Table S1. Effects of cold-pressed cakes in ruminant diet on *in vitro, in vivo and in situ* parameters, growth performance, and FA profile of milk and meat.

Cold-Pressed Cake	Animals		Trial Duration	Inclusion in Diet	Main effects	Reference
	Species	n				
RSC	cows	3	*in situ*	/	RSC in comparison to canola meal had higher rumen effective degradability of CP, NDF and ADF. Soluble CP fraction was higher for RSC, insoluble but potentially degradable fraction was lower, while undegradable fraction was unchanged.	[56]
RSC	ewes (rumen fluid)	2	*in vitro*	27 or 117 g/kg DM	Both examined RSC levels (3% and 6% fat in diet) showed no change in total VFA and methane production in comparison to palm fat. Gas production parameters in the 96h incubation were also unaffected.	[54]
RSC	ewes (rumen fluid)	3	*in vitro*	148 g/kg DM	Replacing palm fat with RSC did not affect DM, OM, CP, NDF, ADF and starch disappearance. Total VFA production, acetate:propionate ratio and methane production were also unaffected. Content of total SFA in rumen effluent was lowered, cis-9, trans-11 CLA was unaffected, while VA was increased.	[55]
RSC	steer calves	140	63[7] + 149[8] days	150 or 300 g/kg DM	Inclusion of RSC (both examined levels) in barley-based diets did not cause any change in ADG or DMI during overall feeding period, whereas higher inclusion level reduced gain to feed ratio. Carcass quality was unaffected. Higher inclusion level increased content of PUFA, n-3, CLA and VA, with no influence on SFA and MUFA content in *pars costalis diaphragmatic* muscle. Concomitant decrease in n-6/n-3 ratio was noticed.	[36]
RSC	dairy cows	40	2 indoor periods (6 months each)	150 g/kg DM	For cows fed RSC (as main protein source) milk yield increased, but contents of milk fat and protein decreased, compared to control diet in which a conventional protein source was used. Content of UFA in milk was increased, including CLA and VA. Negative effects on animal health and fertility were not observed, as well as no problem with off-flavors in milk.	[48]
RSC	dairy cows	4	18 days	0.5 kg crude fat per animal per day	RSC as a fat source in dairy cow diet increased milk yield and fat content, while it decreased protein, lactose and casein content in comparison to soybean meal. Increase of MUFA, PUFA, CLA and VA was observed in milk fat and in its product (cheese).	[59]
RSC	dairy cows	16	24 days	123 g/kg DM	Feeding RSC as a fat supplement was associated with an increase in milk yield and a slight decrease in milk protein concentration, compared to control diet that contained cracked wheat and solvent extracted canola. No change in milk fat and lactose concentration was observed. Increase in dietary fat concentration in dairy cow nutrition successfully reduced enteric CH_4 emissions.	[60]

RSC	dairy cows	58[1] + 35[2]	2 consecutive lactations	735[1] / 580[2] g/kg concentrate DM	Fat-rich RSC as a partial substitute for pelleted wheat and solvent-extracted rapeseed meal decreased milk protein content, while no effect on milk yield, milk fat and lactose content was observed for both indoor and outdoor periods. Supplementation had no effect on body weight, body condition score and plasma indicators of cow metabolic status.	[61]
RSC	dairy cows	58[1] + 35[2]	2 consecutive lactations	735[1] / 580[2] g/kg concentrate DM	Control concentrate was composed of pelleted wheat and solvent-extracted rapeseed meal, which was partially substituted with RSC in experimental diet. Increase in milk MUFA and PUFA, and decrease in SFA content was observed during both indoor and outdoor periods. Content of CLA and VA in milk was increased but only during indoor period.	[82]
RSC	dairy cows	56	90[5] / 275[6] days	100[5] / 66[6] g/kg DM	No significant differences in milk yield and composition were observed by feeding cold-pressed RSC compared with full fat rapeseed.	[62]
RSC	dairy cows	18	10 weeks	234 g/kg concentrate DM	Feeding RSC had no detrimental effects on dairy cows production performance (milk yield and quality) compared to control diet that contained palm kernel meal and hydrogenated palm fat. A diet with RSC affected some microbial taxa at the rumen level, modified the fatty acid biohydrogenation process, which all led to a slight improvement in the milk FA profile. The milk n-6/n-3 ratio was lowered, but there was no increase in beneficial CLA and VA. Better flavor and overall acceptability of milk were noted.	[12]
RSC	dairy sheep	36	38 days	500[3] / 300[4] g/kg as fed concentrate	Inclusion of RSC (both examined levels) instead of soya did not cause any change in milk production and composition. Decrease in milk SFA and increase in UFA was noted for both indoor and outdoor periods. Contents of CLA and VA in milk were moderately increased, but only during indoor feeding.	[66]
RSC	dairy sheep	72	3 weeks	225 g/kg concentrate DM	Replacement of soya by RSC did not compromise animal production parameters of dairy sheep. No differences were found in milk and cheese protein and fat percentages. Milk and cheese from sheep fed RSC showed higher content of UFA and tocopherols. However, concentration of CLA in milk and cheese was unchanged, while VA was increased. Typical sensory attributes of cheese were unchanged.	[67]
RSC	dairy sheep	72	56 days	400 g/kg concentrate DM	Feeding RSC as a replacement for soybean meal and hydrogenated palm fat had no detrimental effects on dairy sheep production performance (milk yield and quality) or curd acceptance. Increase in MUFA, PUFA, n-6 and n-3 was observed in milk, as well as decrease in n-6/n-3 ratio. Potential increase in milk CLA and VA content was observed only when RSC was fed together with tanniferous forage (sainfoin hay).	[68]
HSC	calves[7]/ steers[8]	55[7]/ 51[8]	7[7]/ 9[8] months	1[7] / 0.2 or 1.4[8] kg HSC per animal per day	Inclusion of HSC as a protein feed instead of soybean meal had no influence on liveweight gain in neither calves nor steers. DMI was higher and feed efficiency was lower for HSC supplemented calves diet, while the same was not observed in steers. No changes in carcass measurements were noted. Due to a higher fiber content and a lower starch content rumen function was improved.	[1]

HSC	steers	16	175 (±18) days	0.2 or 1.4 kg HSC per animal per day	Feeding steers with HSC led to an increase in MUFA, CLA and VA content in *M. longissimus dorsi* in comparison to soybean meal, and a decrease in n-6/n-3 ratio. Content of SFA, PUFA, n-6 and n-3 was unaffected by dietary treatment.	[83]
HSC	ewe lambs	48	8 weeks	218 g/kg DM	Inclusion of HSC in barley-based lambs diet did not lead to improvements in growth performance and feed conversion in comparison to control diet without protein supplement. High content of iNDF was observed in HSC, and low digestibility of RUP, which resulted in high amounts of indigestible CP in the feed.	[57]
HSC	ewe lambs	48	8 weeks	218 g/kg DM	Inclusion of HSC in barley-based lambs diet had no effect on *m. longissimus dorsi* content of SFA, MUFA, CLA, VA, n-3 and n-6/n-3 ratio, with the tendency to increase PUFA and n-6, in comparison to control diet without protein supplement.	[84]
HSC	dairy cows	40	5 weeks	143, 233 and 318 g/kg DM	An enhancement of HSC in dairy cow ratio induced an increase in milk yield and a decrease in milk protein and fat concentration. Efficiency of converting dietary CP into milk protein was also decreased. Maximum inclusion level of HSC was 143 g/kg DM based on the highest results obtained for yields of: milk, ECM, milk protein, fat and lactose.	[2]
HSC	dairy sheep	30	10 weeks	213.3 g/kg as fed	HSC as a replacement for sunflower meal increased milk yield and milk fat content, while milk protein content was unaffected. FA profile of milk was enhanced with n-3 FA (notably ALA), CLA and VA, leading to a desired decrease of n-6/n-3 ratio. Notable increase of α-tocopherol in milk fat contributed to preventing lipid oxidation in raw milk.	[49]
HSC	dairy goats	28	45 days	60 and 120 g/kg feed mixture	Feeding goats with HSC as a partial replacement for soybean meal and extruded soybean did not influence milk yield, as well as milk fat and protein content. Higher inclusion level increased milk urea concentration. Increase in capric acid (C10:0) which contributes to the sensory and nutritional properties of goat milk was also observed. However, the transfer of n-3 from feed to milk fat was very low. Haematological and biochemical parameters in lactating goat blood were mostly unaffected by dietary treatments.	[25]
LSC	dairy cows	4	18 days	0.5 kg crude fat per animal per day	LSC as a fat source in dairy cow diet increased milk yield, while it decreased protein, fat, lactose and casein content in comparison to soybean meal. Increase of MUFA, PUFA, CLA and VA was observed in milk fat and in its product (cheese).	[59]
LSC	dairy cows	8	42 days	32, 64, 92 g/kg DM	Gradual replacement of heat-moisture treated RSC with LSC caused a decrease in milk production and milk protein content, while milk fat content was increased. Only minor effects on milk fatty acid profile were observed.	[20]

LSC	dairy cows	16	70 days	213 g/kg concentrate DM	Inclusion of LSC did not lead to improvement in mill yield and milk concentration of protein, fat and casein, when compared to control group that contained RSC. Content of lactose in milk was decreased. Milk content of MUFA, n-6 and n-3 was increased, with no change in SFA and PUFA. Decrease in milk n-6/n-3 ratio was obtained. No negative effects on animal health were observed.	[63]
LSC	dairy goats	16	5 weeks	199 g/kg DM	Supplementation of goat diet with LSC increased milk yield, with no decrease in milk fat content in comparison to control diet that contained extracted rapeseed meal. Content of MUFA and CLA in milk increased, while the SFA (especially C12 to C16) decreased.	[69]
SFC	ewes (rumen fluid)	2	*in vitro*	45 or 225 g/kg DM	Higher inclusion level of SFC (corresponding to 6% fat in diet) resulted in reduced total VFA and methane production, and a trend of fermentation pattern towards lower acetate production in comparison to palm fat. Likewise, only higher inclusion level of SFC resulted in reduced asymptotic gas production.	[54]
SFC	ewes (rumen fluid)	3	*in vitro*	169 g/kg DM	Replacing palm fat with SFC reduced DM, OM, CP, NDF and ADF disappearance, with no change in starch disappearance. Total VFA production, acetate:propionate ratio, as well as methane production were reduced. Content of total SFA in rumen effluent was lowered, while cis-9, trans-11 CLA and VA increased.	[55]
SFC	dairy cows	10	126 days	230 g/kg concentrate DM	Replacement of palm kernel meal and hydrogenated palm fat with SFC caused no negative effects in digestibility, production performance, or milk acceptance. Total content of CLA and VA was increased in milk fat, while n-6/n-3 ratio was unaffected. Feeding SFC induced a decrease in the percentages of C12:0 and C16:0 and an increase of C18:0 in milk fat.	[64]
SFC	dairy sheep	36	38 days	500[3] / 300[4] g/kg as fed concentrate	Inclusion of SFC (both examined levels) instead of soya did not have detrimental effect on milk yield or protein content, while a decrease in milk fat content was observed. Decrease in milk SFA and increase in UFA was noted for both indoor feeding and outdoor grazing. Contents of CLA and VA in milk were increased during both examined periods.	[66]
SFC	dairy sheep	72	56 days	560 g/kg concentrate DM	Feeding SFC as a replacement for soybean meal and hydrogenated palm fat had no detrimental effects on dairy sheep production performance (milk yield and quality) or curd acceptance. Concentration of MUFA, PUFA, n-6, n-3, CLA and VA in milk was increased, but also n-6/n-3 ratio.	[68]
CSC	steers	4	*in vivo*	/	The high oil content of CSC (260 g/kg DM) did not influence microbial activity *in vitro*. Based on *in vivo* digestibility of organic matter and protein, CSC was a superior protein source to sunflower meal or copra meal, and comparable with corn gluten.	[22]
CSC	cows	3	*in situ*	/	Total digestible protein of CSC was in range of linseed meal and soybean meal, but with higher content of ruminally degradable protein. CSC was more ruminally degradable and intestinally digestible than canola meal and DDGS.	[58]

CSC	heifers	110[1]/ 88[2]	78[1]/59[2] days	94[1]/104[2] g/kg DM	CSC as a protein source did not cause any change in ADG and body condition score in heifers in comparison to diet containing DDGS. No adverse effects on reproductive performance were observed.	[52]
CSC	heifers	42	12 weeks	100 g/kg DM	Feeding CSC to growing dairy heifers resulted in similar DMI and ADG compared with linseed meal or DDGS. Gain:feed ratio was similar for CSC and DDGS, and higher for linseed meal. Most of the blood metabolites and metabolic hormones were similar among treatments. Rumen fermentation characteristics were maintained.	[38]
CSC	dairy cows	4	18 days	0.5 kg crude fat per animal per day	Inclusion of CSC led to a decrease in milk yield, as well as protein, fat, lactose and casein content in comparison to soybean meal. However, significantly high quantities of CLA and VA were found in cow's milk and cheese. Increase in milk and cheese MUFA and PUFA was also observed.	[59]
CSC	dairy cows	24	/	50 and 100 g/kg DM	Partial or complete replacement of sunflower meal with CSC did not affect milk yield and milk composition (protein, fat and lactose). FA profile of milk was improved, by increasing PUFA and MUFA content, especially n-6 PUFA. Higher proportion of CLA in milk was observed, especially with higher inclusion of CSC in diet.	[65]
PSC	dairy goat	36	28 days	121 g/kg concentrate	Goat milk yield, as well as milk protein, fat and lactose content were not influenced by inclusion of PSC in diet as a replacement for sunflower meal. Decrease in SFA and increase in MUFA and PUFA was observed in milk fat. Content of n-3 FA was unaffected, while n-6 were increased, leading to a higher n-6/n-3 ratio. Content on CLA was slightly increased.	[15]

[1]—1st year; [2]—2nd year; [3]—indoor period; [4]—outdoor period; [5]—early lactation; [6]—later lactation; [7]—growing diet; [8]—finishing diet; RSC—cold-pressed rapeseed cake; HSC—cold-pressed hempseed cake; LSC—cold-pressed lineseed cake; SFC—cold-pressed sunflower cake; CSC—cold-pressed camelina seed cake; PSC—cold-pressed pumpkin seed cake; DDGS—distillers dried grains with solubles; CP—crude protein; DM—dry matter, OM—organic matter; iNDF—indigestible NDF; RUP—rumen undergradable crude protein; ADG—average daily gain; DMI—dry matter intake; VFA— volatile fatty acids; ECM—energy corrected milk; FA - fatty acids; SFA—saturated fatty acids; MUFA—monounsaturated fatty acids, PUFA— polyunsaturated fatty acids; UFA—unsaturated fatty acids; VA— vaccenic acid; CLA - cis-9, trans-11 conjugated linolenic acid
