# Supplementary material for: Cold-Pressed Oilseed Cakes as Alternative and Sustainable Feed Ingredients: A Review
Source: Foods. 2023 Jan 17;12(3):432. doi: 10.3390/foods12030432 (PMC9914438; doi:10.3390/foods12030432)
Supplement: Supplementary file 1 [file foods-12-00432-s001.zip › Supplementary table S2.pdf]

Supplementary Table S2. Effects of cold-pressed cakes in pig diet on nutrient digestibility and growth performance.

Cold-Pressed Cake	Animals		Trial Duration	Inclusion in Diet	Main Effects	Reference
	Species	n				
RSC	growing pigs	6	14 days	30% as fed	The AID and TTAD of CP were not influenced by dietary inclusion of cold-pressed cakes. The AID of crude fat was higher for diet with HSC and faba bean than for diets with LSC and RSC. Dietary treatment affected the AID of essential AA and non-essential AA (except for phenylalanine, alanine and glycine), with generally slightly lower values for diets with LSC and RSC. Lower AID values were obtained for individual protein feed ingredients than those for the whole experimental diets.	[18]
RSC	growing pigs	7	9 days	44%	The AID and total tract energy digestibility in CPC was higher in heated versus non-heated conditions, and was higher in fast screw speed than in low screw speed. Also the AID of energy of CSC was greater than expeller-pressed rapeseed meal and rapeseed. The DE and calculated NE values of CSC was higher than that in expeller-pressed rapeseed meal.	[16]
RSC	growing pigs	5	9 days	500 g/kg as fed	The RSC contained more EE and less CP and NDF than rapeseed expeller, and also lower CAID and CTTAD of GE and CSID of AA. Porcine *in vitro* DM digestibility was higher for CSC than for rapeseed expeller. Additionally, RSC showed to have lower GE but greater DE value and calculated NE value than rapeseed expeller.	[10]
RSC	growing-finishing pigs	60	64 days	starter—5.5%<br>grower—14%<br>finisher—12%	The animals fed RSC were characterized by lower ADG and higher FCR. The carcasses of pigs fed diets containing RSC had lower lean content than the carcasses of pigs fed toasted soybeans, while backfat thickness and meat quality were unaffected by dietary treatment. RSC with increased oil improved fatty acid profile of backfat, by increasing the concentrations of MUFA and decrease in n-6/n-3 PUFA ratio.	[39]
HSC	growing pigs	6	14 days	28% as fed	The IAD and TTAD of CP were not influenced by dietary inclusion of cold-pressed cakes. The IAD of crude fat was higher for diet with HSC and faba bean than for diets with LSC and RSC. Dietary treatment affected the IAD of essential AA and non-essential AA (except for phenylalanine, alanine and glycine), with generally slightly lower values for diets with LSC and RSC. Lower IAD values were obtained for individual protein feed ingredients than those for the whole experimental diets.	[18]

LSC	growing pigs	6	14 days	24.5% as fed	The IAD and TTAD of CP were not influenced by dietary inclusion of cold-pressed cakes. The IAD of crude fat was higher for diet with HSC and faba bean than for diets with LSC and RSC. Dietary treatment affected the IAD of essential AA and non-essential AA (except for phenylalanine, alanine and glycine), with generally slightly lower values for diets with LSC and RSC. Lower IAD values were obtained for individual protein feed ingredients than those for the whole experimental diets.	[18]
CSC	growing pigs	7	7 days	40%	The SID of CP in CSC-2 was less than the SID of CP in canola meal, but the SID of CP in CSC-1, and CSC-3 was not different from the SID of CP in canola meal. The SID of Lys was not different among all three CSC and canola meal. The SID of AA in CSC was mostly comparable with that of canola meal.	[37]
CSC	growing pigs	n.d.	28 days	37; 74 g/kg as fed	The supplementation of CSC to soy based diets improved feed efficiency but also significantly increased the liver weights. CSC also increased hepatic expression of phase 1 and 2 xenobiotic detoxifying enzymes.	[41]
CSC	fattening and finishing pigs	24	33 days	12%	CSC did not have negative effect on feed intake, feed efficiency or average gain weight. However, CSC modulated cellular immune response by decreasing the protein and gene expression of pro-inflammatory markers, and increased the mRNA expression of antioxidant enzymes in spleen. CSC improved the blood biochemistry profile by decreasing plasma glucose.	[50]
CSC	growing pigs	6	9 days	25%	Multienzyme supplementation at 0.5 g/kg did not affect SID of AA and NE values, and IVDDM for CSC. However, multienzyme at 50 g/kg increased IVDDM of CSC by at least 16%	[23]
CSC	growing pigs	6	2x10 days	200 g/kg as fed	The CP, GE, methionine, lysine, threonine, crude fat, NDF, ADF and glucosinolate concentration of CSC were 21.5 MJ/kg, 381, 18, 6.8, 16.8, 119, 315, 203 g/kg and 36.3 mol/g, respectively. The SID coefficient of CP for CSC was 0.65, whereas the coefficient of SID of lysine, methionine and threonine for SPCC were 0.58, 0.53 and 0.53, respectively. The DE, ME, and calculated NE values of CSC were 17.5, 16.2 and 10.2 MJ/kg, respectively.	[47]
CSC	growing pigs	30	16 days	30% / 10% / 20% as fed	DE, ME, and calculated NE content of CSC fed to growing pigs were 3.755, 3.465, and 2.383 kcal/kg, respectively. Also, additivity of DE, ME, and calculated NE was observed in the mixed diets which contained corn, soybean meal and CSC, pointing out that dietary glucosinolates originating from CS supplementation (up to 30%) did not affect DE, ME, and calculated NE of diets fed to finishing pigs.	[29]

RSC—cold-pressed rapeseed cake; HSC—cold-pressed hempseed cake; LSC—cold-pressed lineseed cake; SFC—cold-pressed sunflower cake; CSC—cold-pressed camelina seed cake; PSC—cold-pressed pumpkin seed cake; CP—crude protein; EE—ether extract; AID—apparent ileal digestibility; SID—standardized ileal digestibility; TTAD—total tract apparent digestibility, CAID—coefficient of apparent ileal digestibility; CSID—coefficient of standardized ileal digestibility; CTTAD—coefficient of total tract apparent

digestibility; IVDDM—in vitro digestibility of dry matter; GE—gross energy; DE—digestible energy; NE—net energy; AA—amino acid; ADF—acid detergent fiber; NDF; neutral detergent fiber ADG—average daily gain; FCR—feed conversion ratio
