# Supplementary material for: Cold-Pressed Oilseed Cakes as Alternative and Sustainable Feed Ingredients: A Review
Source: Foods. 2023 Jan 17;12(3):432. doi: 10.3390/foods12030432 (PMC9914438; doi:10.3390/foods12030432)
Supplement: Supplementary file 1 [file foods-12-00432-s001.zip › Supplementary table S3.pdf]

Supplementary Table S3. Effects of cold-pressed cakes in poultry diet on growth performance, blood biochemistry profile, and FA profile and quality of egg and meat.

Cold-Pressed Cake	Animals		Trial Duration	Inclusion in Diet	Main Effects	Reference
	Species	n				
RSC	Laying hens	216	168 days	5%; 10%; 15%	Feed intake, egg mass production and feed-to-egg mass ratio decreased at 15 % RSC level compared with the 5 and 10 % cake level groups. Increasing dietary level lowered the yolk percentage and increased the egg white percentage. The level of SFA and MUFA decreased, while the content of LA and ALA increased with increasing dietary level.	[19]
HSC	Laying hens	102	4 weeks	50 g/kg; 100 g/kg; 200 g/kg;	The inclusion of HSC did not affect egg production, feed consumption, feed efficiency, or body weight. Egg weight, albumen quality, and the weight of the major egg components were unaffected by HSC. Increasing the amount of HSC in the diet lowered the percentage of palmitic acid in the yolk, and increased the percentages of LA and ALA.	[26]
HSC	Laying hens	216	168 days	5%; 10%; 15%	Feed intake was unaffected by dietary inclusion of HSC. Increasing dietary level lowered the yolk percentage and increased the egg white percentage. The level of SFA and MUFA decreased, while the content of LA and ALA increased with increasing dietary level. HSC with ''linoleic acid oil'' resulted in the highest yolk fat content of this fatty acid.	[19]
HSC	Laying hens	120	10 weeks	20.3%	The inclusion of HSC in the diets of laying hens did not have an adverse effect on the growth performance. The content of ALA, EPA and DHA in egg yolk increased with dietary inclusion of HSC, while the atherogenicity index and cholesterol level were unaffected. The inclusion of HSC was less effective in maintaining the oxidative stability of egg lipids than hemp seed.	[30]
LSC	Laying hens	216	168 days	5%; 10%; 15%	Feed intake was unaffected by dietary inclusion of LSC. Increasing dietary level lowered the yolk percentage and increased the egg white percentage. The level of SFA and MUFA decreased, while the content of LA and ALA increased with increasing dietary level. LSC with ''linolenic acid oil'' resulted in the highest yolk fat content of this fatty acid.	[19]
SFC	Laying hens	1280	54 weeks	26 g/kg	The inclusion of SFC tended to improve FCR, while the dry matter of faeces was decreased in comparison to the control diet. SFC negatively affected hygiene in aviary hens by increasing the proportion of dirty eggs and worsening the foot pad cleanliness.	[27]
CSC	Laying hens	72	7 weeks	10%	The addition of CSC did not exert any detrimental effects on egg weight, FI, or FCR. Egg quality and sensory profile of eggs were unaffected by the addition of CSC. The addition of 10% CSC reduced MUFA level in yolk lipids and significantly increased n-3 PUFA content, in particular ALA, EPA and DHA, compared to the control group.	[87]

CSC	Broilers	196	37 days	50 g/kg; 100 g/kg	CSC depressed the FI, FCR and growth of the broiler chickens, but did not cause any significant enlargement of the thyroid gland, nor were any liver lesions observed. CSC increased the level of n-3 fatty acids in broiler meat, particularly ALA and did not have any adverse effect on the sensory quality of meat.	[33]
CSC	Broilers	384	6 days	120 g/kg; 240 g/kg; 360 g/kg as fed	Increasing dietary level of CSC1 and CSC2 decreased P utilization in broilers. True ileal P digestibility in CSC1 and CSC2 was 35% and 24%, respectively, while true P retention values were estimated to be 15% and 21%, respectively.	[88]
CSC	Broilers	600	20 days	80 g/kg as fed	Feeding the CSC diet did not influence broilers performance parameters or relative weights of lymphoid tissue, except the weight of bursa that decreased. CSC affected plasma lipids profile by decreasing the concentrations of glucose, total cholesterol, high-density lipoprotein cholesterol, and low-density lipoprotein cholesterol. In all lymphoid tissue, the content of n-3 PUFA increased, and the level of n-6 PUFA and n-6/n-3 ratio decreased as the effect of CSC diet.	[51]
CSC	Broilers	456	20 days	100 g/kg	The use of CSC did not have a significant effect on the growth performance of the chickens. CSC supplementation increased the content of n-3 PUFA, especially ALA, but reduced the content of MUFA and n-6/n-3 ratio. CSC slightly worsened the flavor and tastiness of the meat.	[85]
CSC	Broilers	486	21 days	10%	Feeding broiler chickens with camelina cake at 100 g/kg of diet in the second growth period had no negative impact on plasma glucose, triglycerides, total cholesterol and its HDL fraction as well as on concentration of thyroxine and triiodothyronine in plasma.	[89]
CSC	Broilers	240	28 days	3%	CSC supplementation caused an improvement of fatty acids profile of breast meat, by increasing concentrations of PUFA.	[86]

RSC—cold-pressed rapeseed cake; HSC—cold-pressed hempseed cake; LSC—cold-pressed lineseed cake; SFC—cold-pressed sunflower cake; CSC—cold-pressed camelina seed cake; PSC—cold-pressed pumpkin seed cake; SFA—saturated fatty acids; MUFA—monounsaturated fatty acids; PUFA—polyunsaturated fatty acids; LA - linoleic acid; α-linolenic acid; ALA; EPA - eicosapentaenoic acid; DHA - docosahexaenoic acids; FCR—feed conversion ratio; FI—feed intake
